# Navigating the Spectrum of Conventionality: Toward a New Model of Creative Thinking

**DOI:** 10.3390/jintelligence11020021

**Published:** 2023-01-18

**Authors:** Kristin Lansing-Stoeffler, Nola Daley

**Affiliations:** ACT, Inc., Iowa City, IA 52240, USA

**Keywords:** creativity, creative thinking, innovation, assessment, education

## Abstract

Current conceptualizations of creative thinking focus primarily on the measurement of creative thinking for the purpose of identifying creative thinking proficiency. We propose a conceptualization that includes a framework and assessments that focus on the measurement *and* learning of creative thinking and innovation skills. Our conceptualization involves an understanding that innovation is a critical application of creative thinking and that the process of creative thinking that leads to innovation can be performed intentionally and explicitly. In this paper, we put forth a process model for creative thinking and innovation that focuses on an expanded set of cognitive and social skills and processes that facilitate the navigation of the spectrum of conventionality. The process model includes the conventional thinking skill, which serves as not only a foundational skill for understanding and navigating the spectrum of conventionality, but also facilitates the reliable measurement of creative thinking and innovation by supporting the generation of a response pool that represents the full spectrum of conventionality for use in scoring. We explore the advantages of this model and how it addresses some of the challenges presented by current creative thinking conceptualizations and assessments. Finally, we explore the implications of implementing this process model for education.

## 1. Introduction

As the global economy transforms into a knowledge-based society with an enhanced focus on information and innovation, success will depend on the ability of global citizens to have a strong foundation of working effectively with knowledge (Drucker 1993; Sawyer 2012; Schwab 2017). Working effectively with knowledge requires thinking both critically and creatively. Creative Thinking and Innovation skills have been identified as essential for success in the modern classroom and modern society (Organisation for Economic Co-operation and Development (OECD 2018); Schleicher 2020; United Nations Children’s Fund (UNICEF 2022); World Economic Forum (WEF 2016)).

Thinking skills that focus on the generation of ideas that both defy conventions and maintain value as appropriate for the given context fall into the realm of what is often referred to as creative thinking (Kaufman and Sternberg 2010; Mumford et al. 2012; Sternberg 1999). The value of these skills in education and the workforce is driven by their ability to fuel the innovations of the future. While creative thinking skills can be applied in many ways that improve society, through the arts and humanities for example, it is the power of this skillset to facilitate innovation, generate new solutions, products, and ideas that raise it to the level of importance heralded by groups deeming it an essential skill for the future (OECD 2018; Schleicher 2020; UNICEF 2022; WEF 2016). Applying creative thinking skills intentionally with the goal of innovation is facilitated by a conceptualization of creative thinking and innovation as a specific set of skills and processes.

Models of dual processing distinguish between fast, automatic, and unconscious thinking or slow, deliberative, controlled and conscious thinking can be represented as dual-process models (Evans 2009; Sloman 1996). Our conceptualization of creative thinking adopts a similar dual-process model to explain the process of generating creative ideas. Specifically, as with previous dual process models, we see these unconscious and conscious modes of thinking as both competing and cooperating in the process of generating creative ideas (Allen and Thomas 2011). However, only the deliberate/conscious process can be learned and applied with intention while the benefits of the automatic processes provide an unconscious supporting role. These unconscious and conscious modes of creative thinking could be represented as a spontaneous route and an intentional route. The spontaneous route appears automatic in that an unconventional idea arrives as a spark of creativity or a ‘stroke of genius’. The intentional route is more deliberate and takes into account the understanding that ideas exist on a spectrum of conventionality that can be navigated intentionally with skills and processes. 

For the first route, the spark of creativity is often preceded by incubation, or facilitated by mind-wandering (Martindale 1999; Radel et al. 2015) or brainstorming (Rawlinson 2017). This spontaneous route is the creative thinking process that is supported by a shower, a good nap, a long walk outdoors, or a group brainstorming session to spur the spontaneous arrival of an unconventional idea. Individuals that struggle to generate unconventional ideas on this route have little recourse and may benefit from a more explicit route. The learning and development of skills and strategies to navigate this route (e.g., incubation, mind-wandering, brainstorming) provide few opportunities for development beyond practice or tacit knowledge. 

Beyond the spontaneous route, we propose a route that is more intentional and can be applied intentionally toward an anticipated outcome. Our conceptualization is based on the understanding that ideas exist on a spectrum of conventionality that can be navigated intentionally with skills and processes, as well as an understanding of the intrapersonal factors that facilitate those processes. At one end of the spectrum are ideas that are common, or conventional, in their theme or approach. Conventional ideas can be defined as themes or approaches frequently reported by individuals in response to a given stimulus. At the other end of the spectrum of conventionality are unconventional ideas, ideas that occur infrequently or rarely as themes or approaches to the given stimulus. This statistical infrequency criterion allows us to evaluate the uniqueness of a response as a deviation from the collection of responses (Guilford 1950). For an individual to cultivate the ability to generate ideas that are at the unconventional end of the spectrum an understanding of the spectrum, and the features and qualities of the ideas that determine their placement on the spectrum, can serve to facilitate the creative thinking and innovation process. 

The outcome of both of these routes, the generation of unconventional ideas, can be measured using creative thinking assessments; however, the intentional route can be intentionally taught, learned, and reliably applied. The intentional route also leaves room for the strategies of the spontaneous route (e.g., incubation, mind-wandering, brainstorming) to be applied, perhaps even applied more effectively. In this paper we present a construct model designed to support the learning and measurement of creative thinking and innovation skills in a way that improves on the reliability, and scalability of previous conceptualizations.

## 2. Conceptualizations of Creative Thinking

Conceptualizations of creative thinking involve both the identification and definition of the skills involved with creative thinking (frameworks) and methods of measurement designed to elicit evidence of those skills (assessments). Definitions of creative thinking vary dramatically by field and organization. A review by Meusburger (2009) noted over 100 different definitions across fields and organizations, varying by the context in which creative thinking is applied. Current conceptualizations of creative thinking, however, coalesce in their focus on the role of creative thinking in generating ideas that defy conventions while maintaining value as appropriate for the given context (Kaufman and Sternberg 2010; Mumford et al. 2012; OECD 2022; Sternberg 1999). As definitions of creative thinking and their corresponding frameworks vary based on the context in which creative thinking is being applied, we are focusing on conceptualizations of creative thinking within the context of education and measurement. Conceptualizations of creative thinking within the context of education and measurement fall into two main approaches, those designed for self- and other-report surveys and those designed for product-based assessments. Each of these approaches has advantages and disadvantages.

### 2.1. Self- and Other-Report Assessment of Creative Thinking

Self-report assessments of creative thinking focus on creative achievements or personality scales as an indication of proficiency with creative thinking. Assessments such as the Creative Achievement Questionnaire (CAQ) ask participants to reflect on their own creative achievements across 10 domains (Carson et al. 2005). Participants check items describing their accomplishments. Each domain contains 8 items weighted with a score from 0 to 7. The CAQ is scored based on the weighted total of achievements across those domains. While easy to administer and score, the insights provided by the assessment are limited to the identification of those who are already creative thinkers. Insights for those developing their creative thinking and innovation are limited to understanding where additional opportunities for creative achievement might exist.

Other-report assessments of creative thinking use scales of teacher ratings to allow teachers to rate the creativity of their students. Teacher rating scales of students’ creative thinking are a widely used approach for evaluating students for entrance to talented and gifted programs including the Scales for Rating the Behavioral Characteristics of Superior Students (Renzulli et al. 2010), the Scales for Identifying Gifted Students (Ryser and MacConnell 2004), the Gifted Rating Scales (Pfeiffer and Jarosewich 2003), and Having Opportunities Promotes Excellence scale (Gentry et al. 2015). The scales ask teachers to report on their perceived estimates of student characteristics in areas related to creative thinking. Similar to self-report, other-reporting measures can be convenient and inexpensive options for evaluating students’ creative thinking. Other-reporting measures are also less susceptible the biases that are inherent in self-report surveys. The accuracy of teacher ratings using these scales for predicting students’ creative thinking, however, is low (Gralewski and Karwowski 2013). Other disadvantages stem from the limitations of the perception of ‘others’ to reflect the actual proficiency due to the role that the ego might play (John and Robins 1993), and, in the case of creative thinking specifically, racial or ethnic bias (Peters and Pereira 2017).

In addition to direct measures of creative thinking, personality scales (both self and other reported) measure personality traits believed to be correlated with creative thinking capacity (Costa and McCrae 2008). Questionnaires that focus on aspects of the Big Five Inventory, such as openness to experience, ask participants to reflect on the degree to which they agree with a statement that is aligned to a specific personality trait. Scoring indicates the degree to which a participant exhibits a high or low level of that trait. While the CAQ provides insights regarding an individual’s creative achievement, inventories such as the Big Five provide insights regarding an individual’s creative capacity or tendencies. This information could be useful for the identification of those with creative thinking potential, for individuals; however, the assessment may provide clues for expanding ones’ own creative capacity through improved openness or understanding of their risk tolerance, for example.

The advantages of self- and other-reporting methods lie mostly in their low cost and ease of administration as paper/pencil or online surveys. However, these methods are primarily designed to identify those who are already creative thinkers (or identify those who have a greater tendency for creative thinking). As such, these methods offer limited value in terms of providing specific insight for how individuals can improve their Creative thinking skills. In addition to this main limitation, other limitations of self-report of personality measures are rooted in the social desirability to provide a response that results in a positive reflection of self and the ease of exaggeration or masking of authentic behaviors (Garcia and Gustavson 1997; Northrup 1997; Viswesvaran and Ones 1999; Ziegler et al. 2012). Limitations are also influenced by the contextual nature of the reporting in which the reflection of a participant may be influenced by the emotional state at the time of completing the survey or specific life experiences.

### 2.2. Product-Based Assessment of Creative Thinking

Product-based assessments require individuals to construct a response. The most commonly used product-based tests of creative thinking in education are arethe Torrance Test of Creative Thinking (TTCT; Torrance 1966, 1990) and variants such as the Abbreviated Torrance Test for Adults (ATTA; Goff 2002), as well as assessments by Guilford (1967) and Wallach and Kogan (1965) (Long and Wang 2022). The OECD also developed a Creative Thinking assessment for PISA 2022 which was administered in over 60 countries. Generally, product-based assessments of creative thinking focus on fluency, flexibility, originality, and elaboration skills. These skills are generally elicited using product-based tasks that allow for a range of opportunities for expression and a range of stimulus (e.g., verbal, figural). The product-based response format allows participants to construct their responses, and generally include domain agnostic stimuli to limit the influence of prior knowledge on performance. These components allow for a broad range of both expression of ideas for participants and opportunities for inspiration; however, Long and Wang (2022) found that most of these tasks rely on alternative use tasks in which participants brainstorm to identify alternative uses for common objects and that those objects were limited to mainly a brick, box, or knife (e.g., Lee and Therriault 2013).

Additional limitations of product-based assessments of creative thinking stem from the item design and scoring. Prompts for product-based creative thinking tasks often offer participants little direction regarding the skill that is being elicited by the task (e.g., “name things with wings”). This approach provides limited direction regarding the skill that is being elicited, limiting the participants’ ability to focus on fluency, flexibility, or originality—the skills on which their response will be scored. Alternatively, the student may be prompted to focus on one creative thinking skill (e.g., “provide as many ideas as possible” for fluency, or “provide an idea that not many people would think of” for originality) with their response being scored across the full range of skills and criteria (e.g., Torrance 2018). Providing direction that elicits a single skill and then scoring that response against multiple criteria of which the student was not prompted creates a challenge for the participant to provide a response that demonstrates their proficiency with the skills that are ultimately being measured. Deviating from the design and scoring method of scoring multiple skills from a single item response, the PISA 2022 Creative Thinking assessment included items designed to measure and report a score for a single skill (OECD 2019). While this provides a student with the opportunity to demonstrate their proficiency with a specific skill, the use of a single stimulus for multiple items and skills may introduce an effect in which the item itself is functioning to reduce functional fixedness and facilitate the generation of unconventional ideas on subsequent items related to that stimulus. 

An additional limitation of scoring for product-based assessment is that the pool of responses that are used to determine the frequency of themes, which in turn determines what qualifies as conventional or unconventional for scoring, are responses that are generated for a creative thinking assessment in which participants are explicitly encouraged to provide creative ideas. It could be argued that the pool of responses represent an over-sampling of unconventional ideas. It could also be argued that participants will refrain from including ideas that they consider to be conventional. When those conventional ideas are included as responses, they will be infrequent and therefore scored as unconventional ideas. For example, if US students are asked to provide creative boys names that begin with ‘M’ they will likely avoid common names such as Mike, Matt, or Mark making these responses infrequent in the response pool and therefore qualifying them as creative for scoring purposes.

The scoring of product-based responses also has significant limitations. Human-scoring of student responses requires training, a level of expertise, and inherently involves a degree of subjectivity (Long and Wang 2022; Silvia et al. 2008). Inconsistencies also exist among the level of expertise required, the number of raters per item, and the thresholds for inter-rater reliability. Perhaps most problematic for the scoring of creative thinking assessments are the inconsistencies and range of criteria for scoring skills common across assessments as well as the range of inclusion of skills and the weight given to those skills for scoring (Long and Wang 2022). The challenge of scoring creative thinking at an international scale was achieved by the 2022 PISA Creative Thinking assessment through multiple extensive coder trainings, training materials, and query services provided to address coder queries (OECD 2023 in press). Advancements in the scoring of product-based responses have been made using artificial intelligence and machine learning for creative thinking tasks (Forthmann and Doebler 2022; Buczak et al. 2022); however, the application of these techniques has been primarily limited to divergent thinking tasks.

## 3. Creative Thinking and Innovation: Framework

Our conceptualization of creative thinking builds on these prior conceptualizations with an enhanced focus on the skills and processes that support learning as well as measurement. Creative Thinking and Innovation skills are considered vital skills for success in academics and the workforce (Greenstein 2012; Tan 2000). This value makes their teaching and measurement an important inclusion in the classroom (Robinson 2001). Research demonstrates that creative thinking and innovation skills can not only be learned effectively (Amabile 1996; Kaufman and Beghetto 2009; Scott et al. 2004), but that their inclusion in learning in the classroom can contribute to gains in student achievement (Akpur 2020; Anwar and Aness 2012; Gralewski and Karwowski 2012; Gregory et al. 2013; Huang et al. 2017; Sebastian and Huang 2016; Schacter et al. 2006) and improve school performance (Sternberg 2003). Beyond academic performance, training in creative thinking and innovation might also serve to improve creative self-efficacy (Mathisen and Bronnick 2009; Perry and Karpova 2017), and an improve attitudes toward risk-taking (Perry and Karpova 2017). Additionally, research shows that increased creative self-efficacy corresponds with increased creative performance (Tierney and Farmer 2011). The development of the creative thinking and innovation skills can be facilitated by a framework designed to provide insights that support that development.

Our Creative Thinking and Innovation (CTI) construct conceptualizes creative thinking and innovation as a process as well as an outcome. Framing creative thinking and innovation as a process requires an understanding of the skills that support the development of unconventional ideas in a way that allows us to identify the skills lacking in students who are not proficient at generating unconventional ideas. Framing creative thinking and innovation as an outcome requires a focus on the applied value of this capability for both education and the workforce. With this framing in mind, our framework defines creative thinking and innovation as: the skills and processes involved with the generation of ideas that are unconventional, original, or innovative.

**Figure 1:**
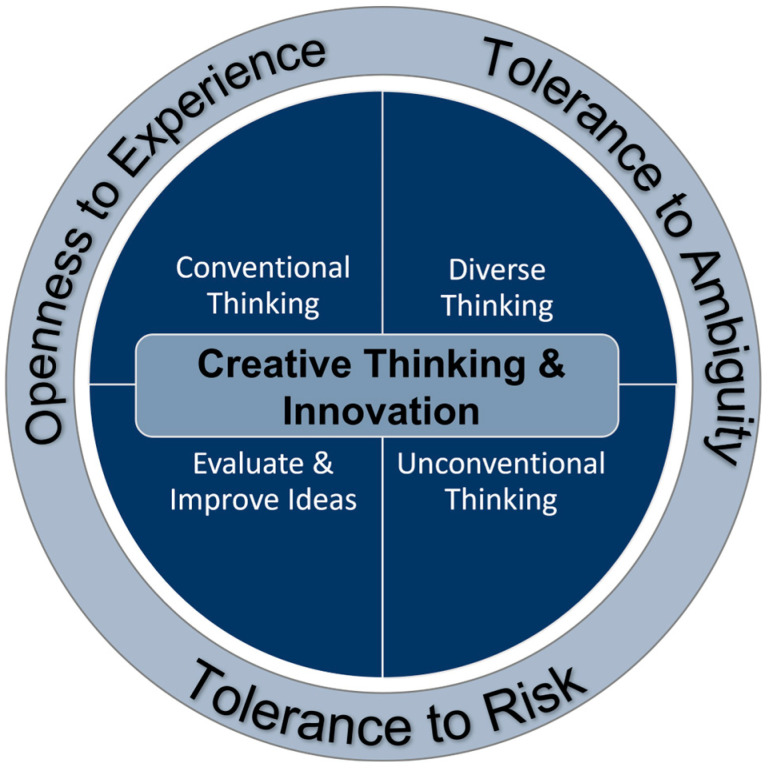
Creative Thinking and Innovation Framework.

Our CTI framework identifies four skills and three traits that support the generation of ideas that are unconventional, original, or innovative. The four skills include conventional thinking, diverse thinking, unconventional thinking, and evaluate and improve ideas. The three traits include openness to experience, tolerance of ambiguity, and risk tolerance (Figure 1).

### 3.1. CTI: Cognitive Skills

Our skill definitions (Table 1) call out both the ability to identify and generate skills. This allows for a range of proficiency to be identified through measurement and expands our ability to cultivate this skillset at scale by facilitating measurement. At the lowest level of proficiency, learners may be able to only identify conventional or unconventional ideas or improvements, or to identify ideas that are qualitatively different from other ideas, with learners building on this knowledge to move toward the generation of ideas.

#### 3.1.1. Conventional Thinking

The conventional thinking skill is focused on the identification and generation of conventional ideas in compliance with given criteria. Responses to a request for an idea that is original, unique, or innovative will fall on a spectrum of conventionality. At one end of the spectrum are ideas that are common in their theme or approach. The commonness of the ideas is reflected by the frequency of the theme or approach in the pool of individual responses. At the other end of the spectrum of conventionality are unconventional ideas, ideas that occur infrequently or rarely as themes or approaches. This statistical infrequency criterion allows us to evaluate the uniqueness of a response as a deviation from the collection of responses (Guilford 1950). We propose that for an individual to cultivate the ability to generate ideas that are at the unconventional end of this spectrum an understanding of the spectrum itself and the features and qualities of the ideas that determine their placement on the spectrum can serve to facilitate the creative thinking process. 

Responses in the form of ideas, solutions, or artefacts can be placed on the spectrum of conventionality based on the categorization of the idea as embodying a theme or approach. These categorizations of themes and approaches are determined by the features that define them (Baer 2016; Cropley 2006; Lucas and Spencer 2017). It is these features and criteria that ultimately define their placement on the spectrum of conventionality. These features also provide insights and opportunities for innovative expansion. For example, if a student is asked to come up with a creative song and, after incubating, they are still struggling to generate an unconventional idea, they can begin the process of identifying conventional songs and the features that make them conventional. Features may include 4/4 time signature, tempo kept by a drum, a voice singing a melody, and instruments providing the instrumental structure. With identification of these features, learners now have the option to explore each of these features as opportunities to diverge from the conventions. In this sense, conventional ideas can be the seeds of unconventionality for individuals looking to engage in creative thinking as an explicit process. The outcomes of the conventional thinking skill include an understanding of conventions and their features in a way that facilitates an individuals ability to expand beyond those conventions.

#### 3.1.2. Diverse Thinking

The diverse thinking skill is focused on the identification and generation of diverse ideas in compliance with given criteria. Our definition of this skill goes beyond ideational fluency, in which value is placed on the quantity of ideas produced, to focus instead on ideational flexibility, or the quality of ideas produced and the degree to which they diverge qualitatively from each other in theme, approach, etc. (Guilford 1956). Ideational flexibility correlates highly with ideational fluency (Hébert et al. 2002; Torrance 1969, 1972) and while ideational fluency is a valuable creative thinking strategy based on the tendency for conventional ideas to come first, followed by unconventional ideas (Beaty and Silvia 2012; Lubart et al. 2003), this strategy does not function as a skill that can be improved, but rather a strategy that can be employed or practiced. Learners can be directed to generate more ideas to improve their ability to perform well on ideational fluency tasks. To improve ideational flexibility, learners benefit from understanding how ideas differ qualitatively and begin to explore those differences as opportunities to navigate the spectrum of conventionality.

Diverse thinking defined as fluency, focused on the quantity of ideas, supports the creative thinking process as a strategy that helps the mind move beyond functional fixedness (Amabile 1983). Functional fixedness, or an inability to consider an object/idea beyond its intended application, may serve as a barrier to the generation of an unconventional idea (Amabile 1983; Duncker 1945). Diverse thinking as ideational flexibility could support expansion of ideas beyond what is common or familiar by making the process of navigating the spectrum of conventionality explicit. Prompting learners for ideational flexibility elicits higher flexibility scores (Runco and Okuda 1991). For example, a student asked to name different courses that begin with the letter ‘A’ could respond with a long list of advanced math classes (Advanced Algebra, Advanced Statistics, Advanced Calculus, Advanced Algebra II, Advanced Geometry, etc.). While there are differences in the math content in each of these classes, they all fit under the broad category of math classes. Alternatively, a student could respond with a list of classes that differ significantly qualitatively from each other in content (e.g., Art, Astronomy, Algebra), demonstrating a broader range of divergence. Explicitly asking a student for ideas that are as different from each other as possible can elicit ideas that are higher in ideational fluency (Runco and Okuda 1991). Framing the diverse thinking skill as focused on the generation of qualitatively divergent ideas allows us to support learners in not just generating a high quantity of ideas (which all may be similar) to focusing on the features and qualities of those ideas. This, in turn, supports individuals’ abilities to generate ideas that expand on the spectrum of conventionality to the unconventional end of the spectrum. Challenges to the generation of diverse ideas could result from a lack of understanding of the themes or approaches for ideas on the spectrum of conventionality and their features. The outcome of the diverse thinking skill includes an understanding of the qualities of ideas (in theme, approach, etc.) in a way that supports the ability to generate multiple ideas that diverge qualitatively from each other.

#### 3.1.3. Unconventional Thinking

While the generation of diverse ideas can support the navigation of the spectrum of conventionality with new placements on the spectrum, these ideas, though different from each other, could still be common in their theme or approach. For example, Algebra and Art, are two different courses that begin with the letter ‘A’, but these could also be considered common responses. The unconventional thinking skill is focused on the identification and generation of ideas that fall at the unconventional end of the spectrum, outside of social norms and occur infrequently or rarely as themes or approaches in responses (Guilford 1950). For a student to provide an unconventional course that begins with the letter ‘A’, the student would benefit from an understanding of what a conventional courses that begin with the letter ‘A’ might be, and evaluate the conventionality of their ideas against that understanding.

Beyond the uniqueness of an idea, appropriateness is also considered an essential component of an unconventional idea (Amabile 1983; Kaufman and Baer 2012; Runco and Jaeger 2012; Sternberg 1999). Definitions of appropriateness range from ‘usefulness’ (Mayer 1999) to ‘effectiveness’ (Runco and Jaeger 2012) and allude to criteria that range from assigning value based on context-specific criteria to evaluating ideas for plausibility. Our approach to appropriateness as a criterion for unconventional ideas is focused on the minimal criteria that to be appropriate an idea needs to be on-topic and on-task. This criterion was also used for the 2022 PISA Creative Thinking Assessment (OECD 2018). This frees us from evaluating ideas for assertions of value as well as the inherent limitations presented by evaluations of plausibility rooted in a quickly evolving world of advancements.

The generation of unconventional ideas is enabled by a conducive environment, sufficient motivation, sufficient knowledge or skills and a process that leads to the generation of an unconventional idea (Amabile 1983). The challenges to demonstrating this skill are both internal and external to the individual (Amabile 1983; Amabile and Pratt 2016; OECD 2022; Sternberg and Lubart 1991, 1995). Functional fixedness, the high value placed in society on conventional ideas, and intrapersonal factors such as openness to new ideas, risk aversion, and psychological safety are all factors that impact the generation of unconventional ideas. 

While incubation and the process of allowing the mind to wander might support the seemingly spontaneous generation of unconventional ideas (Martindale 1999; Radel et al. 2015), this can be attributed to incubation as an opportunity for ‘forgetting’, allowing the individual to move beyond functional fixedness (Smith 1995) and to allow for the unconscious to move beyond the conscious motivation to create logic or order (Csikszentmihalyi 1996). Cognitive, or functional, fixedness can impede the creative thinking process due to a tendency of individuals to fixate on the typical or conventional functions (Adamson 1952; Agogué et al. 2014a, 2014b; Duncker 1945). This bias can function as a familiar rut of sorts allowing us to find common or correct solutions, but can challenge our ability to ‘get off the beaten path’ to less familiar and less conventional territory (Adamson 1952; Agogué et al. 2014a, 2014b; Cassotti et al. 2016; Duncker 1945; Purcell and Gero 1996; Smith et al. 1993). Amabile (1983) notes divergent thinking as a tool that also helps the mind move beyond functional fixedness.

The value placed on conventional ideas (DeCoker 2000; Nickerson 2010) contributes to a culture of risk-aversion when it comes to the generation of unconventional ideas (Amabile 2012; Nickerson 2010; Wong and Niu 2013). This also points to a need for an environment of psychological safety in which individuals feel comfortable, if not encouraged, to contribute unconventional ideas (Hu et al. 2018; Zhou and Pan 2015). As with many skills, the willingness to contribute or demonstrate that skill will be influenced by the environment in which the skill is being elicited. Cultivating an environment that takes these factors into consideration facilitates the contribution, and accurate representation, of an individual’s ability to generate unconventional ideas.

The unconventional thinking skill allows individuals to explicitly demonstrate their ability to identify or generate unconventional ideas. Success with this skill implies the ability to understand the spectrum of conventionality, the themes and approaches that define that spectrum, and the ability to identify or generate ideas that occur with statistical infrequency at the unconventional end of the spectrum without extending off the end of the spectrum as an idea that is implausible or inappropriate.

#### 3.1.4. Evaluate and Improve Ideas

The evaluate and improve skill is focused on the identification and generation of ideas that iterate and improve on given ideas to improve the unconventionality of those ideas, essentially to make an existing idea more creative. In the real world, the creative thinking processes are often applied with the intention of making an existing idea more creative. The initial idea can be placed on the spectrum of conventionality and this skill is focused on making improvements to that idea that move the idea toward the unconventional end of the spectrum. Beyond the unconventionality of the improvement, fidelity to the original idea is also an essential component. This criterion requires that the improvement does not simply replace the original idea but maintains some fidelity to, or preserves the essence of, that idea in that it is still recognizable in the final product. For example, a student might be given a short poem with a conventional title and be asked to generate a new, more creative, title for the poem that incorporates the words from the original title. Students are not asked to come up with their own unconventional title, rather to work with the constraints provided by the original title to create something more unconventional.

Improving the unconventionality of an idea is facilitated by first evaluating that idea to identify the features that define, or limit, its conventionality (Martindale 1999; Radel et al. 2015). Evaluation is used throughout the creative thinking process to evaluate ideas against the criteria that define the task (Beghetto et al. 2014; Sawyer 2012). The improvement of the idea is facilitated by not only understanding the features that define or limit its conventionality, but also the ability to incubate or generate qualitatively diverse solutions and understand the conventionality of those solutions in a way that leads to an unconventional improvement. 

The application and value of this skill in the real world are seen most notably in the roles of editors and critics. An editor can evaluate and improve the creativity of writing without being an expert on the topic or writing the manuscript. A food critic can provide insights to improve a dish without being a master chef. A movie critic can craft a plot twist without being a director, actor or producer. Expertise in these areas can provide enhanced insights and improvements, but as we know from the concept of functional fixedness this can also be a limitation, providing credence for the value of diverse perspectives. The outcome of this skill includes the ability to evaluate the features that define a convention in a way that facilitates the generation of an improvement in unconventionality that respects and retains elements of the original idea.

### 3.2. Creative Thinking and Innovation: Traits

The challenges to effectively engaging in the creative thinking and innovation process are both internal and external to the individual (Amabile 1983; Amabile and Pratt 2016; OECD 2022; Sternberg and Lubart 1991, 1995). While there is a wide range of environmental (classroom) enablers (e.g., educational approaches, school and classroom climate, cultural norms and expectations), and social factors (e.g., task motivation, collaboration, domain readiness, etc.) (OECD 2019) influencing the creative thinking process we focus on factors that are within the student’s control. Engagement in the creative thinking process is enabled by a range of intrapersonal factors, or traits. The inclusion of these traits in the creative thinking and innovation process facilitates the understanding of these factors as internal barriers to, or facilitators of, creative thinking with the intention of improving an individual’s ability to fully engage in the creative thinking and innovation process and apply these skills in the real world.

#### 3.2.1. Openness to Experience

Openness to experience refers to the degree to which an individual is inquisitive, imaginative, and curious about unusual ideas or people (Ashton and Lee 2007). Openness to Experience has an established relationship with creativity and creative thinking (Dollinger et al. 2004; Feist 1998; King et al. 1996) as the desire to explore alternative and unconventional solutions requires a high-level of openness (McCrae 1987). As with the other social skills, an individual’s awareness of their own openness to experience can support individuals in addressing this as a potential barrier to the generation of unconventional ideas and the creative thinking and innovation process. A lack of openness to experience is considered to be expressed as a lack of curiosity, avoidance of creative pursuits, and aversion to ideas that seem unconventional (Ashton and Lee 2007). Low openness to experience would clearly present a barrier to the generation of unconventional ideas. For example, a lack of curiosity would potentially limit diverse idea generation and engagement in process to generate unconventional ideas. An aversion to ideas that are unconventional also presents obvious limitations to their generation. 

#### 3.2.2. Tolerance of Ambiguity

Tolerance of Ambiguity refers to the degree to which an individual perceives ambiguous situations as desirable (Stanley Budner 1962). If what is conventional is comfortable, as the creative thinking process expands beyond what is conventional this can create an environment in which a tolerance for ambiguity could facilitate the creative thinking process (Zenasni et al. 2008). Individuals with a low tolerance for ambiguity may be drawn to categorization, certainty, and the familiar (Bochner 1965), creating conditions in which an individual is more likely to engage in functional fixedness. An individual’s understanding of their own tolerance for ambiguity can facilitate one’s own ability to address this as a barrier to the creative thinking and innovation process.

#### 3.2.3. Risk Tolerance

While tolerance of ambiguity relies on the perception of an ambiguous situation as desirable, the degree to which an individual perceives a situation as anxiety-inducing or dangerous depends on their risk aversion. Activities that involve intentional engagement with tasks that entail novelty or danger in a manner that is sufficient to create anxiety in most people are considered risk taking activities (Levenson 1990). Research also suggests that even those confident in their creative ability may require high levels of intellectual risk taking to develop creative behavior (Beghetto et al. 2021). Risk taking in Creative Thinking has been looked at through the lens of those engaging in tasks embedded in domains considered to be creative (e.g., artistic or literary) and a high tolerance for risk was found to be correlated with more creative ideas (Lubart and Sternberg 1995). The generation of an unconventional idea depends on the risk taking involved with breaking from the conventional, functional fixedness (Prabhu 2011). Risk taking can also be approached in a decontextualized, domain-agnostic, way to understand an individual’s general propensity for risk taking (Nicholson et al. 2005).

Similar to openness to experience, a higher tolerance for risk may facilitate an individual’s ability to engage with the creative thinking process with a mindset that does not see breaking from conventions as a risky endeavor. A low risk tolerance may result in increased anxiety involved with the creative thinking process when seen as a risky behavior and result in risk avoidance behaviors in which the individual avoids or disinvests from the creative thinking process.

Possible limitations of this model. Our creative thinking and innovation model does not account for all skills or all traits that influence an individuals ability to effectively engage with the creative thinking and innovation process, but aims to focus on key elements that can be used to inform development and identify progress with proficiency. A wide range of additional factors, including the classroom environment and motivational factors, also influence the creative thinking and innovation process (Cremin and Chappell 2021); however, these are beyond the scope of insights that can be provided through assessment. We anticipate that assessments designed from this model will provide valuable insights that individuals and teachers can utilize to inform development of this skillset. Future pre- and post-research as well as longitudinal research will be valuable to inform the effectiveness of this model.

## 4. Creative Thinking and Innovation: Measurement

As Creative Thinking and Innovation skills have been identified as essential for success in the modern classroom and modern society (OECD 2018; Schleicher 2020; UNICEF 2022; WEF 2016), ensuring that learners are gaining proficiency with these skills is facilitated by their measurement. The methods for the measurement of creative thinking generally include instruments that focus on either self- or other-reporting or product-based assessment.

The limitations of current assessments, noted above, create challenges for scalability and inclusion in the classroom (Long and Wang 2022). To answer these challenges and to facilitate the development of this essential skillset, we propose specific assessment design recommendations based on the use of the assessment. These recommendations fall into two categories: recommendations for learning and development of creative thinking and innovation skills with a focus on scalable, low-stakes assessments designed to provide learners with insights that support the development of skills; and recommendations for higher-stakes assessment focused on smaller administrations that allow for more authentic demonstrations of skill and intensive scoring.

To facilitate the development of this skillset in the classroom, we propose measuring skills that can be developed to improve creative thinking and innovation proficiency. We propose focusing on skills that support the understanding and navigation of the spectrum of conventionality: conventional thinking, diverse thinking, unconventional thinking, and evaluate and improve. These align with the traditional creative thinking skills of flexibility, originality, and elaboration. We include the skill of conventional ideas to improve learners understanding of the role of conventions in generating unconventional ideas and navigating the spectrum of conventionality. The inclusion of the conventional thinking skill also serves to expand the response pool for establishing conventionality to include the full spectrum of conventionality, which has the potential to create significant impacts for scoring.

Building on these guidelines, the conventional thinking skill could be measured by prompting students to provide common ideas or ideas many other people might think of, or by having students identify the most common idea in a set of ideas. The diverse thinking skill could be measured by prompting students to provide ideas that are as different from each other as possible, or by having students identify an idea among a set of ideas that is as different as possible from a given idea. The unconventional thinking skill could be measured in a similar way to the conventional thinking skill, prompting students to provide an idea that not many other people would think of, or identifying the most uncommon idea in a set of ideas. The evaluate and improve skill could be measured by prompting students to improve on the creativity of a given idea or to identify an idea in a set of ideas that most improves on the creativity of an idea.

We also recommend the inclusion of surveys with the assessments to address the traits that influence an individual’s ability or willingness to engage in the creative thinking process: openness to experience, tolerance of ambiguity, and risk tolerance. Several scales currently exist for the assessment of personality traits, for example the HEXACO Model of Personality Structure Personality Inventory (Ashton and Lee 2009), and NEO Personality Inventory (Costa and McCrae 2008). We recommend ensuring the surveys used are age-appropriate in terms of length (e.g., short/long scale) and language. Individual’s awareness of their own tendencies with these skills can support individuals in understanding the role these skills play in navigating the spectrum of conventionality and addressing these as a potential barriers to the generation of unconventional ideas and engagement in the creative thinking and innovation process.

While product-based assessments allow students to fully demonstrate their proficiency with a skill, the requirements for scoring make these item types difficult to score at scale (Long and Wang 2022). Training for teachers to score at the classroom level introduces additional feasibility, subjectivity, and comparability issues (Long and Wang 2022; Silvia et al. 2008). For the classroom we propose that selected response items can be built using population-specific (e.g., age, grade) response pools that reflect the full spectrum of conventionality through the inclusion of items that measure both conventional and unconventional ideas. Selected response items alleviate challenges related to the subjectivity and scalability of scoring while providing learners with valuable insights into their skill proficiency. We also propose the measurement of a single skill per item in which students are explicitly prompted for the skill being measured, and the use of a wide range of domain-agnostic stimulus. Ideally, an assessment designed for the classroom with the above principles in mind would provide students with insights into their proficiency with each of the creative thinking and innovation skills along with the role that those skills play in the creative thinking and innovation process, facilitating the development of a creative thinking and innovation skillset. Feedback provided to students regarding the traits involved with the creative thinking and innovation process would also provide students with the opportunity to reflect on their own personal traits and how those traits might inhibit or facilitate their engagement in the creative thinking and innovation process.

Creative thinking and innovation skills have the potential to be significant differentiators in academia and the workforce. These skills help students stand out among their peers and help workers contribute to the advancement of their industries. Colleges and universities have recognized the value of this impactful skill set in transforming content knowledge into innovative and potentially world-changing solutions. As a result, colleges and universities increasingly highlight these skills as differentiators for admissions (Adobe 2020; Pretz and Kaufman 2017). To support leveraging strengths in creative thinking and innovation for higher-stakes purposes, such as college admissions and career applications, we propose the use of constructed response items that allow for a full range of expression and inspiration. As with classroom assessments, we propose the measurement of a single skill per item in which students are explicitly prompted for the skill being measured, and the use of a wide range of domain-agnostic stimulus. We also propose the use of stimulus in a way that does not introduce an effect in which the item itself is functioning to reduce functional fixedness and facilitate the generation of unconventional ideas on subsequent items relate to that stimulus. This can be achieved by the use of a unique stimulus for each item or the strategic dispersion of stimulus throughout the assessment. Items designed to measure the critical thinking skills could follow a similar format to the items designs (prompts) described for K12 with a focus on the generation of responses (constructed responses) rather than the identification of ideas. The subjectivity and comparability issues involved with scoring constructed response items could be alleviated by leveraging artificial intelligence and machine learning advancements in this space (Forthmann and Doebler 2022; Buczak et al. 2022). As with the classroom assessment, it is essential that response pools reflect the full spectrum of conventionality through the inclusion of items that measure both conventional and unconventional ideas.

Ideally, an assessment designed for higher-stakes purposes with the above principles in mind would provide students with insights into their strengths with each of the creative thinking and innovation skills which could then be shared with institutions and programs that seek students with a creative thinking and innovation skillset. This would both broaden the range of strengths that students could share with institutions, beyond academics, and enhance the ability of institutions to connect with students that demonstrate proficiency with the creative thinking and innovation skillset and the potential to fuel the innovation of their programs.

## 5. Discussion

While current conceptualizations of creative thinking focus primarily on the measurement of creative thinking for the purpose of identifying creative thinking proficiency, we have proposed a conceptualization of creative thinking that focuses on the measurement and learning of creative thinking and innovation skills. A conceptualization of creative thinking and innovation skills that is designed to both support the development of a creative thinking and innovation skillset and provide insights into proficiency with the skillset benefits from the inclusion of a collection of skills that contribute to the development and understanding of unconventional ideas. Those skills benefit from being defined at a level of detail that supports the design of assessments to measure those skills with the intention of providing insights to inform student proficiency and the further development of those skills. A conceptualization of creative thinking and innovation skills that is designed to both support the development of a creative thinking and innovation skillset and provide insights into proficiency with the skillset also benefits from the inclusion of traits that contribute to an individuals ability and willingness to engage in the creative thinking and innovation process. While there are many factors that influence the ability and willingness of an individual to effectively engage in the creative thinking and innovation process, we focus our conceptualization on factors that are within the students’ control.

Our expanded conceptualization involves an understanding that innovation is a critical outcome and application of creative thinking and that the process of creative thinking and innovation can be learned and performed intentionally and explicitly. This conceptualization is dependent on an understanding of the spectrum of conventionality and the tools required to navigate that spectrum. 

In this paper, we put forth a process model for creative thinking and innovation that focuses on the cognitive and social skills and processes that facilitate the navigation of the spectrum of conventionality. We outlined the relevant skills: conventional ideas, diverse ideas, unconventional ideas, and evaluate and improve ideas and how they support the creative thinking process and navigation of the spectrum of conventionality. This also includes a new skill: conventional ideas, which serves as not only a foundational skill for understanding and navigating the spectrum of conventionality, but also a skill that facilitates the reliable measurement of creative thinking and innovation by supporting the generation of a response pool that represents the full spectrum of conventionality for use in scoring.

We explored the advantages of this model and how it addresses some of the challenges presented by traditional creative thinking conceptualizations and assessments. To support the development of creative thinking skills in the classroom, we propose item and scoring design solutions that leverage the advantages of product-based assessment and advancements in artificial intelligence and machine learning to support both the learning and high-stakes measurement of creative thinking and innovation. To support the development of creative thinking skills in the classroom, while alleviating challenges related to subjectivity and scalability, we propose that the use of selected response items can be built using population-specific (e.g., age, grade) response pools that reflect the full spectrum of conventionality through the inclusion of items that measure both conventional and unconventional ideas. Selected response items alleviate the challenges of scoring while providing learners with valuable insights into their skill proficiency both effectively and efficiently. We also propose the measurement of a single skill per item in which students are explicitly prompted for the skill being measured, and the use of a wide range of domain-agnostic stimulus.

To support the measurement of creative thinking skills for higher-stakes purposes, we propose leveraging constructed response items that allow for a full range of expression and inspiration. As with classroom assessments, we propose the measurement of a single skill per item in which students are explicitly prompted for the skill being measured, and the use of a wide range of domain-agnostic stimulus. To address the subjectivity and comparability issues involved with scoring constructed response items, we propose leveraging artificial intelligence and machine learning for the evaluation of responses (Forthmann and Doebler 2022; Buczak et al. 2022) and the identification of scoring themes. As with the classroom assessment, it is essential that response pools reflect the full spectrum of conventionality through the inclusion of items that measure both conventional and unconventional ideas.

Our intention is that our process model will provide new opportunities to facilitate both the learning and measurement of creative thinking and innovation skills from the classroom to the boardroom. Planned future research in this area includes assessments for both low- and high-stakes applications to provide reliability and validity evidence of the effectiveness of the approach. It is important to expand this research to include culturally diverse populations. Longitudinal studies would also facilitate the identification of long-term impacts of the inclusion of creative thinking in the classroom.

## Figures and Tables

**Table 1:** Creative Thinking and Innovation Framework Skills and Definitions.

Skill	Definition
Conventional Thinking	The identification and generation of conventional ideas in compliance with given criteria.
Diverse Thinking	The identification and generation of diverse ideas in compliance with given criteria.
Unconventional Thinking	The identification and generation of unconventional or unique ideas in compliance with given criteria.
Evaluate and Improve Ideas	The identification and generation of ideas that iterate and improve on given ideas to improve creativity.

## Data Availability

Not applicable.
